# Machine learning powered ellipsometry

**DOI:** 10.1038/s41377-021-00482-0

**Published:** 2021-03-12

**Authors:** Jinchao Liu, Di Zhang, Dianqiang Yu, Mengxin Ren, Jingjun Xu

**Affiliations:** 1 The Key Laboratory of Weak-Light Nonlinear Photonics, Ministry of Education, School of Physics and TEDA Applied Physics Institute, Nankai University, Tianjin, 300071 China; 2 College of Artificial Intelligence, Nankai University, Tianjin, 300071 China; 3 Collaborative Innovation Center of Extreme Optics, Shanxi University, Taiyuan, Shanxi 030006 China

**Keywords:** Optical spectroscopy, Optical metrology

## Abstract

Ellipsometry is a powerful method for determining both the optical constants and thickness of thin films. For decades, solutions to ill-posed inverse ellipsometric problems require substantial human–expert intervention and have become essentially human-in-the-loop trial-and-error processes that are not only tedious and time-consuming but also limit the applicability of ellipsometry. Here, we demonstrate a machine learning based approach for solving ellipsometric problems in an unambiguous and fully automatic manner while showing superior performance. The proposed approach is experimentally validated by using a broad range of films covering categories of metals, semiconductors, and dielectrics. This method is compatible with existing ellipsometers and paves the way for realizing the automatic, rapid, high-throughput optical characterization of films.

## Introduction

Ellipsometry is a contactless, nondestructive, widely used optical technique for measuring the optical constants (refractive index *n* and extinction coefficient *κ*) of materials^1^. It is self-evident that optical constants provide the fundamental basis for designing and manufacturing optical devices ranging from cell phone cameras to sophisticated photonic integrated circuits^2–4^. Furthermore, it is widely recognized that the optical constants, acting as “fingerprints” of the materials, provide a means to inspect the macroscopic and microscopic properties of the substances, such as electronic structures^5–7^, doping concentration^8,9^, and surface properties^10^. Such success was demonstrated by Drude more than 100 years ago when monitoring the formation of a contaminant layer on a freshly cleaved crystal^11^. Currently, ellipsometers have prevailed in both scientific labs and industrial companies. Any improvements in ellipsometry would benefit the broad fields of science and technology.

**Fig. 1:**
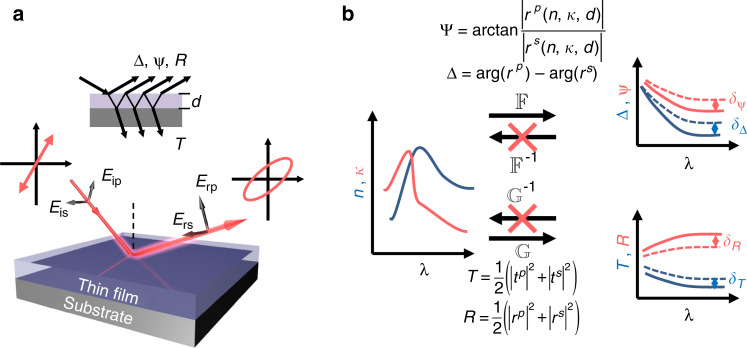
Principle of *R*, *T* assisted ellipsometry. **a** In ellipsometry, light *E*_*i*_ is obliquely incident onto a thin film with unknown (*n*, *κ*, *d*), which is on a substrate with known optical constants. Multiple beam interference occurs in the film, as shown in the inset. The orthogonal polarization components (*E*_*p*_ and *E*_*s*_) have unequal reflection coefficients *r*_*p*_ and *r*_*s*_, leading to elliptically polarized reflection *E*_*r*_. *p* and *s* refer to polarizations with electric fields parallel and normal to the plane of incidence, respectively. **b** The ellipsometric angles Ψ and Δ are defined by, tan Ψ e^iΔ^ = *r*_*p*_/*r*_*s*__,_ as denoted by ${\Bbb F}$. The total transmittance *T* and reflectance *R* are calculated by averaging the contributions from the *p*- and *s*-polarizations, denoted by ${\Bbb G}$. The physical process from (*n*, *κ*, *d*) to (Ψ, Δ, *R*, *T*) has been well studied and can be modeled accurately as functions ${\Bbb F}$ and ${\Bbb G}$. On the other hand, the inverse, i.e., analytically inferring (*n*, *κ*, *d*) from (Ψ, Δ, *R*, *T*), is generally impossible, and iterative fitting techniques have therefore been developed to numerically search a set of (*n*, *κ*, *d*) that best fits the experimental data. Solid lines denote experimentally measured (Ψ, Δ, *R, T*), dashed lines present results recovered by the tentative (*n, κ, d*) solution

Ellipsometry is a process of inferring (*n*, *κ*) by a set of measured ellipsometric angles (Ψ, Δ), which relates to the amplitude ratio and phase difference between the complex reflection coefficients of parallel (*p*) and perpendicular (*s*) polarizations, respectively (as indicated in Fig. 1). Mathematically, such a procedure belongs to the category of an “inverse problem”, which aims to derive the “causes” from the “results”^12–15^. For the inverse ellipsometric problem, especially when samples consist of one or more films on a substrate, analytical solutions generally do not exist, and “regression data fitting” techniques have been developed that iteratively find a set of optical parameters that best fit the observations.

As the inverse ellipsometric problem is usually ill-posed^16–18^, the existing fitting techniques still rely on certain forms of trial-and-error learning. In conventional fitting techniques, human–expert intervention is indispensably required to provide a good initial “guess” for the target sample properties to achieve fitting convergence. Traditionally, various dispersion equations have been developed and adopted to describe the materials and generate the initial values for fitting, such as the Cauchy and Sellmeier models^19,20^, the Forouhi–Bloomer^21,22^ and Tauc–Lorentzian equations^23^, or the Drude-Lorentz model^24,25^. Users must be highly experienced with these optical models to make proper choices. In particular, it is often required to combine multiple dispersion models to precisely describe a sample, which leads to too many correlated fitting parameters and makes the initial guessing even more difficult. Without a good initial solution to start with, the traditional fitting methods can hardly converge to the correct solution, and the process will have to start over again. The overall process inevitably becomes tedious and extremely time-consuming. Furthermore, for films with unknown thickness *d*, two equations from Ψ and Δ are generally no longer sufficient to uniquely recover the three unknowns of (*n*, *κ*, *d*) simultaneously, and a single pair of (Ψ, Δ) may lead to multiple solutions of (*n*, *κ*, *d*) (see Fig. S1 in supplementary information)^26,27^. Fortunately, it has been proven that by supplementing (Ψ, Δ) data with auxiliary measurement information from transmission (reflection) measurements^28,29^, interference enhancement^30^, or ambient changing^31^, etc., the above mathematical ambiguity can be eliminated, and a unique solution of (*n, κ, d*) can be produced^27^. However, the simultaneous analysis of the complemented ellipsometric measurements remains challenging, as the additional information amplifies the weakness of the traditional techniques and makes the fitting too complicated to be practical.

Inventing a new ellipsometric analysis technique working in a “single-click” manner without the need for human (expert) intervention while showing superior analysis performance to traditional techniques would be a major step towards intelligent and fully automatic ellipsometry for in situ and automatic material measurements and diagnostics. For instance, an artificial neural network (ANN) has been adopted as a data pre-processor to guess an initial (*n, κ, d*) value for the subsequent regression fitting process^32^. This method was shown to be very fast. However, because it only allows one single trial without “closed-loop” feedback and auto-adjustment, the method shows a risk of fitting failure caused by a “bad” initial guess from the ANN.

Recently, artificial intelligence, particularly deep learning^33^, has achieved unprecedented performance in a variety of tasks, including visual recognition^34–36^, natural language processing^37^, machine translation^38^, etc. It has also been successfully applied to solve challenging problems in physics^39,40^, chemistry^41,42^, and biology^43^. In these applications, deep neural networks excel in modeling a real-world physical or chemical process and are flexible to function as building blocks of a framework to solve challenging problems in unconventional ways. Typical examples include the inverse design of nanophotonic particles^44^, enhancing the sensing ability with a learned integrated sensing protocol^45^, identifying the different phases of matter^46^, searching for exotic particles in high-energy physics^47^, solving inverse problems in computational imaging^48^, and acting as a new calculation tool to solve sophisticated quantum problems^49^.

In this paper, we propose a machine learning based approach to solve the ellipsometric problem by deep neural network-driven iterative learning (denominated as “SUNDIAL”). We supplement the ellipsometric measurements with intensity-based transmission *T* and reflection *R* spectroscopies to enrich the data and reduce the illness (ambiguity) of the inverse problems. Using SUNDIAL, the (Ψ, Δ) and (*R*, *T*) spectra are analyzed simultaneously, and unique solution sets (*n*, *κ*, *d*) for films are successfully obtained. We further experimentally validate our SUNDIAL method by using a broad range of material films covering the categories of metals, semiconductors, and dielectrics. Our approach is compatible with the present configuration of ellipsometers and can be used directly in existing commercially available products. This novel fully automatic approach based on machine learning paves the way for realizing automatic, rapid, high-throughput optical characterization of films and can be greatly beneficial for real-time quality monitoring in repeatable high-precision film manufacturing.

## Results

Solving the inverse ellipsometric problem relies on a thorough understanding of the forward physical processes. The principle of ellipsometry is based on deciphering the material properties from the changes in the light polarization reflected at oblique angles. The physical description for the forward process in ellipsometry was well developed approximately two centuries ago when Malus discovered the “doubly refractive” like behaviors for light obliquely impinged onto a material^50^. The mathematical description of such anisotropic behaviors was accomplished afterward by Fresnel using a group of equations known now as “Fresnel’s formulae”^51^. After that, Airy further proposed multiple-beam interference formulae to calculate the reflection (*r*) and transmission (*t*) coefficients of film-covered surfaces^52^. Thus, the (Ψ, Δ) and (*R*, *T*) from the samples can be analytically and uniquely determined for a known group of (*n*, *κ*, *d*) from the films as follows:

1 $$\begin{array}{*{20}{c}} {\left\{ {\begin{array}{*{20}{l}} {{\Bbb F}:\;\tan {\mathrm{{\Psi}}}e^{i{\mathrm{{\Delta}}}} = \frac{{r_p\left( {n,\;\kappa ,\;d} \right)}}{{r_s\left( {n,\;\kappa ,\;d} \right)}}} \hfill \\ {{\Bbb G}:T = \frac{1}{2}\left( {\left| {t_p} \right|^2 + \left| {t_s} \right|^2} \right),\;R = \frac{1}{2}\left( {\left| {r_p} \right|^2 + \left| {r_s} \right|^2} \right)} \hfill \end{array}} \right.} \end{array}$$

On the other hand, the analytical solution of the inverse process, i.e., the direct translation of the data (Ψ, Δ) and (*R*, *T*) back to (*n*, *κ*, *d*), is impossible for films (indicated in Fig. 1b) due to the transcendental nature of Airy’s formulae. Thus, the ellipsometric problem becomes ill-posed and very challenging to solve^16–18^.

Mathematically, SUNDIAL aims to solve the following optimization problem:

2 $$\begin{array}{*{20}{c}} {\begin{array}{*{20}{r}} \hfill {\left( {n^ \ast ,\;\kappa ^ \ast ,\;d^ \ast } \right) = {\mathrm{arg}}\;{\mathrm{min}}_{n,\;\kappa ,\;d}\left[ {\left. \gamma \right\|{\Bbb F}\left( {n,\;\kappa ,\;d} \right) - \left( {{\mathrm{{\Psi}}},\;{\Delta}} \right)||_2} \right.}\\ \hfill {\left. { + \left( {1 - \gamma } \right)\left\| {\Bbb G} \right.\left( {n,\;\kappa ,\;d} \right) - \left( {R,\;T} \right)||_2} \right]}\end{array}} \end{array}$$

where $\left\| \cdot \right\|_2$ denotes the Euclidean norm between ${\Bbb F}$(*n*, *κ*, *d*) and (Ψ, Δ) in the ellipsometric angle space, as well as between ${\Bbb G}$(*n*, *κ*, *d*) and (*R*, *T*) in the intensity spectroscopic data space. *γ*∈[0,1] is chosen to balance the weights of (Ψ, Δ) and (*R*, *T*) in the optimization process, and we choose 0.5 here to equilibrate the contributions from the two terms.

**Fig. 2:**
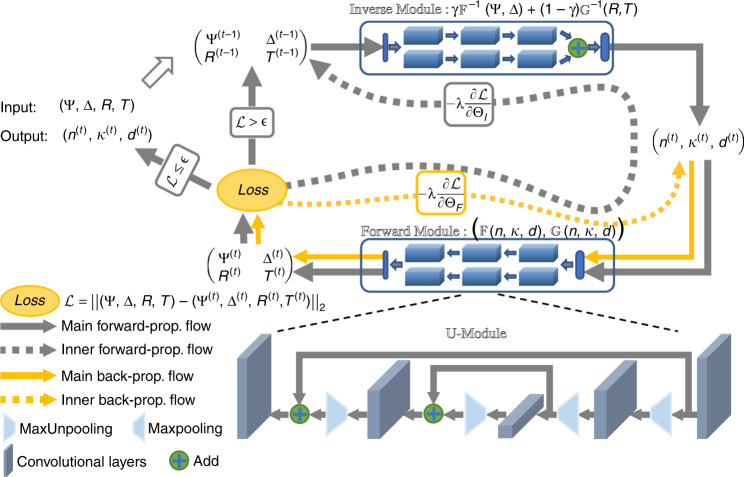
SUNDIAL for ellipsometric analysis. This method consists of inverse and forward neural modules (encircled by rectangles). The inverse module is trained to perform $\gamma {\Bbb F}^{ - 1} + \left( {1 - \gamma } \right){\Bbb G}^{ - 1}$, and the forward module is trained to be surrogates for the functions ${\Bbb F}$ and ${\Bbb G}$. The modules are joined together in a loop manner, as indicated by the gray arrows. A novel iterative inference strategy based on stochastic gradient descent, indicated by the loops enclosed by gray and yellow arrows, is proposed to allow training on analytically generated simulated data and inference on real-world experimental data

Solving Eq. (2) needs a sophisticated optimization toolkit that conceptually includes mechanisms for candidate solution generation, criteria for solution evaluation, and an iterative refinement method. Following this recipe and powered by machine learning, we present below the SUNDIAL approach, which is based on deep neural network brimming with domain/ellipsometric knowledge learned from both offline and online training and is able to solve Eq. (2) efficiently. Specifically, this method takes (Ψ, Δ, *R*, *T*) as the input and automatically outputs a unique (*n*, *κ*, *d*) solution. SUNDIAL admits a novel iterative optimization framework with deep neural networks as the core building blocks. This approach consists of two neural modules, namely, inverse and forward modules (encircled by rectangles in Fig. 2, details of input/output refer to Table I in supplementary information), which are joined together in a loop manner. Conceptually, after proper training, the inverse module performs $\gamma {\Bbb F}^{ - 1} + \left( {1 - \gamma } \right){\Bbb G}^{ - 1}$ and generates candidate solutions (*n*^(t)^, *κ*^(t)^, *d*^(t)^). The forward module is trained to be surrogates for the forward functions ${\Bbb F}$ and ${\Bbb G}$ and serves as a criterion for evaluating candidate solutions. Both modules are implemented as carefully designed convolutional neural networks with stacked residual U-modules.

To acquire domain/ellipsometric knowledge, SUNDIAL is first trained offline on a large amount of simulated data, which may significantly deviate from the real-world ellipsometric data. The simulated dataset includes 6240 pairs of (Ψ, Δ, *R*, *T*) and (*n*, *κ*, *d*) created analytically using the forward modeling functions of ${\Bbb F}$ and ${\Bbb G}$ based on (*n*, *κ*) of 200 different materials from the Palik and Sopra databases^53,54^, and the *d* is varied between 10 and 300 nm with a step of 10 nm for each material (5 nm step is further adopted for lossy materials for *d* below 100 nm, such as gold and silver). To bridge the gap between training on the simulated data and inferring on the real-world data, we propose a novel iterative inference strategy based on stochastic gradient descent, as indicated by the loops enclosed by gray and yellow arrows in Fig. 2. Unlike the conventional inference of computing outputs by a single forward pass on neural networks, which is doomed to fail in our case, the proposed strategy allows neural modules to continue to adapt online on real-world data until a satisfactory solution is obtained. Specifically, given a set of real-world data (Ψ^(0)^, Δ^(0)^, *R*^(0)^, *T*^(0)^), the inverse module computes an initial solution of (*n*^(0)^, *κ*^(0)^, *d*^(0)^). We immediately train the forward module to approximate ${\Bbb F}$ and ${\Bbb G}$ well within a small neighborhood of (*n*^(0)^, *κ*^(0)^, *d*^(0)^) and compute how well the solution (*n*^(0)^, *κ*^(0)^, *d*^(0)^) can reconstruct the ellipsometric data (Ψ^(0)^, Δ^(0)^, *R*^(0)^, *T*^(0)^). The error/residual is then back-propagated through both modules, and the trainable weights in the inverse module are updated. The updated inverse module will generate a better solution (*n*^(1)^, *κ*^(1)^, *d*^(1)^). This procedure repeats until (*n*^(t)^, *κ*^(t)^, *d*^(t)^) reconstructs (Ψ^(0)^, Δ^(0)^, *R*^(0)^, *T*^(0)^) sufficiently well.

**Fig. 3:**
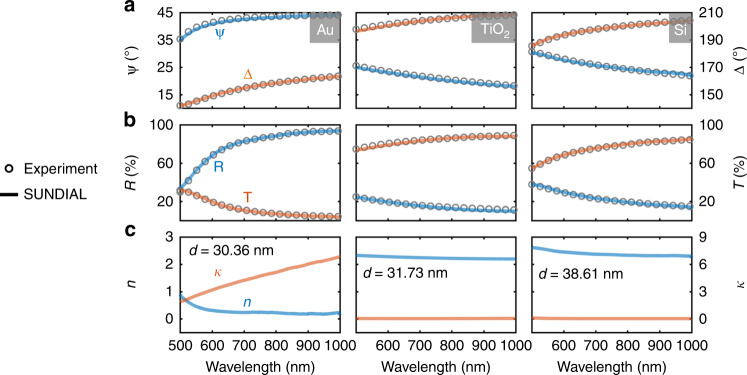
Results of SUNDIAL on Au, TiO_2_, and Si. **a**, **b** Measured Ψ, Δ, *R*, and *T* spectra are presented by empty circles. Model-generated spectra, drawn as solid lines, are calculated using (*n*, *κ*, *d*) by the SUNDIAL method and forward functions ${\Bbb F}$ and ${\Bbb G}$. Each column corresponds to a material. **c** The (*n, κ*, *d*) retrieved by SUNDIAL

We demonstrate the performance of SUNDIAL in experiments using a series of thin films covering categories of metals, dielectrics, and semiconductors. All the films are prepared on fused quartz substrates by thermal evaporation, sputtering, or plasma-enhanced chemical vapor deposition (PECVD) techniques (see methods). Figure 3 exemplifies the results of gold (Au), titanium dioxide (TiO_2_), and silicon (Si) films. The empty circles in Fig. 3a, b indicate the measured (Ψ, Δ, *R*, *T*) data. By feeding these experimental data into SUNDIAL, the dispersions of (*n*, *κ*) and the thickness *d* are computed and outputted automatically, as shown in Fig. 3c. We calculate the (Ψ, Δ, *R*, *T*) curves (solid lines in Fig. 3a, b) following the ${\Bbb F}$ and ${\Bbb G}$ functions based on the derived (*n*, *κ*, *d*), which overlap well with the experimental results. The results of more materials are given in Fig. S3 of the supplementary information, all of which show good agreement between the calculated and measured (Ψ, Δ, *R*, *T*) data and validate our SUNDIAL approach.

**Fig. 4:**
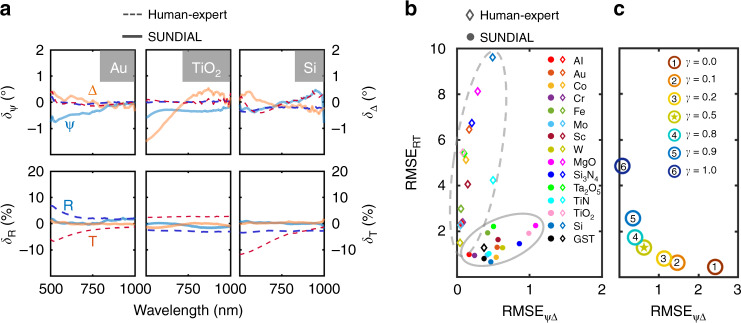
Comparison of SUNDIAL with the traditional fitting technique. **a** Residual on three typical materials of SUNDIAL (solid lines) and the traditional fitting technique (dashed lines). Both methods fit (Ψ, Δ) very well, while the traditional fitting method shows a significant error in fitting (*R*, *T*). **b** RMSE of the compared methods on more tested materials. SUNDIAL (solid dots) significantly outperformed the traditional fitting method (empty diamonds) in terms of overall accuracy. RMSE_ΨΔ_ = RMSE_Ψ_ + RMSE_Δ_, and RMSE_RT_ = RMSE_R_ + RMSE_T_. **c** Effect of *γ*. Experiments with different *γ* values are conducted, and the RMSEs are shown here. Tungsten (W) is used here as an example

To quantitatively compare the performance of our SUNDIAL method with the traditional fitting technique (commercial EP4Model software as an example, Fig. S2), the residual (*δ*) and root mean squared error (RMSE)^31^ values are computed. *δ* is defined as the difference between the forward model (${\Bbb F}$ and ${\Bbb G}$) calculated and the experimentally measured curves: $\delta _S = S^{{\mathrm{cal}}} - S^{{\mathrm{exp}}}$, where *S* is Ψ, Δ, *R*, or *T*. The RMSE is calculated as:

3 $${{\mathrm{RMSE}} = \sqrt {\frac{{\mathop {\sum }\nolimits_{i = 1}^N \left( {S_i^{{\mathrm{cal}}} - S_i^{{\mathrm{exp}}}} \right)^2}}{N}}}$$

where *N* is the number of wavelength points included in each spectrum. Smaller *δ* and RMSE values indicate better matches between the model generated and the experimental data and thus better solutions. The EP4Model software only fits ellipsometric data (Ψ, Δ), while (*R*, *T*) data act as complements to reduce the fit ambiguity and choose the most suitable (*n*, *κ*, *d*) solution branch. The resulting model-generated Ψ and Δ curves agree well with the experimental curves, as demonstrated by the near-zero *δ*_Ψ_ and *δ*_Δ_ values in Fig. 4a and Fig. S4; however, the *δ*_*R*_ and *δ*_*T*_ curves show significant deviation from zero. On the other hand, SUNDIAL is capable of fitting (Ψ, Δ) and (*R*, *T*) simultaneously, leading to near-zero *δ* values for all four curves, as shown by the solid lines in Fig. 4a and Fig. S4. Figure 4b gives the RMSE of (Ψ, Δ), and (*R*, *T*) for different materials by SUNDIAL and the traditional fitting technique. The RMSE could be influenced by the value of γ, as illustrated in Fig. 4c. It can be seen that this is a typical scenario of two-objective optimization^55^. If γ is set to 1, then only (Ψ, ∆) takes effect, while if we set *γ* = 0, then (*R*, *T*) dominates the optimization process. For other *γ* values, both the contributions from (Ψ, ∆) and (*R*, *T*) are considered. In this study, we choose 0.5 to equilibrate the contributions of the two terms. The SUNDIAL results (solid dots and encircled by a solid ellipse) are much closer to the origin than the traditional technique (empty diamonds and encircled by a dashed ellipse), which means that the SUNDIAL optimizes both (Ψ, Δ) and (*R*, *T*) and balances them well. In terms of fitting all four spectra, SUNDIAL significantly outperforms the traditional technique. Furthermore, it is worth mentioning that on a few materials, SUNDIAL has a slightly larger RMSE on (Ψ, Δ) and a significantly smaller RMSE on (*R*, *T*) than the traditional technique. This result occurs due to the inconsistency of the sampling positions for (Ψ, Δ) and (*R*, *T*), for example, and the optimal solution of (*n*, *κ*, *d*) can only be achieved when all four spectra are optimized and well balanced. To eliminate such inconsistency, one may have to update the ellipsometers to realize the in situ comprehensive (Ψ, Δ, *R*, *T*) measurements.

## Discussion

In conclusion, we have proposed a machine learning based approach for solving ill-posed ellipsometric problems in an unambiguous and fully automatic manner. Benefitting from the deep neural networks’ superiority of achieving excellent performance and flexibility as building blocks to solve unconventional tasks, the proposed approach can learn human/ellipsometric knowledge to guide the inverse optimization process and therefore avoid the need for human–expert intervention; therefore, it is more convenient to use. Furthermore, thanks to the simultaneous analysis of additional data (*R*, *T*) along with the traditional ellipsometric data (Ψ, Δ), SUNDIAL is able to mitigate the problem of *(n, κ, d*) ambiguity that traditional fitting techniques suffer from. Such a machine learning powered method is compatible with existing ellipsometers and paves the way for realizing the automatic, rapid, high-throughput optical characterization of the films. This approach is beneficial for real-time in situ quality monitoring for the high-precision repeatable fabrication of layered structures. Our SUNDIAL approach, as a versatile machine learning framework for solving ill-posed inverse problems, can also be extended to other optical measurement techniques.

## Materials and methods

### Data preparation

Our film samples included 15 different materials, namely, Al, Au, Co, Cr, Fe, Mo, Sc, W, MgO, Si_3_N_4_, Ta_2_O_5_, TiN, TiO_2_, Si, and Ge_2_Sb_2_Te_5_ (GST). These samples were fabricated on fused quartz substrates by different techniques: Al, Au, Cr, and GST by sputtering; Co, Fe, Mo, Sc, W, MgO, Ta_2_O_5_, TiN, TiO_2_, and Si by thermal evaporation; and Si_3_N_4_ by PECVD. The ellipsometric Ψ and Δ data were measured in the visible to near-infrared spectral range using a commercial spectroscopic ellipsometer (Imaging Ellipsometer EP4, Accurion Inc., Goettingen, Germany), and the incident angle *θ* was fixed at 50°. The reflection and transmission spectra under normal incidence were measured by a spectrometer (Spectrophotometer U-4100, Hitachi Ltd., Tokyo, Japan).

## Supplementary information

Supplementary information for Machine learning-powered ellipsometry
