# Supplementary material for: Machine learning powered ellipsometry
Source: Light Sci Appl. 2021 Mar 12;10:55. doi: 10.1038/s41377-021-00482-0 (PMC7952555; doi:10.1038/s41377-021-00482-0)
Supplement: Supplementary file 1 — Supplementary information for Machine learning-powered ellipsometry [file 41377_2021_482_MOESM1_ESM.pdf]

Supplementary information for "Machine learning powered ellipsometry"

Jinchao Liu†,[1,2] Di Zhang†,[1] Dianqiang Yu,[1] Mengxin Ren,[1,3,a)] and Jingjun Xu[1,b)]

[1)] *The Key Laboratory of Weak-Light Nonlinear Photonics, Ministry of Education, School of Physics and TEDA Applied Physics Institute, Nankai University, Tianjin 300071, China*

[2)] *College of Artificial Intelligence, Nankai University, Tianjin 300071, China*

[3)] *Collaborative Innovation Center of Extreme Optics, Shanxi University, Taiyuan, Shanxi 030006, China*

[a)] Electronic mail: ren_mengxin@nankai.edu.cn

[b)] Electronic mail: jjxu@nankai.edu.cn

I. MORE RESULTS

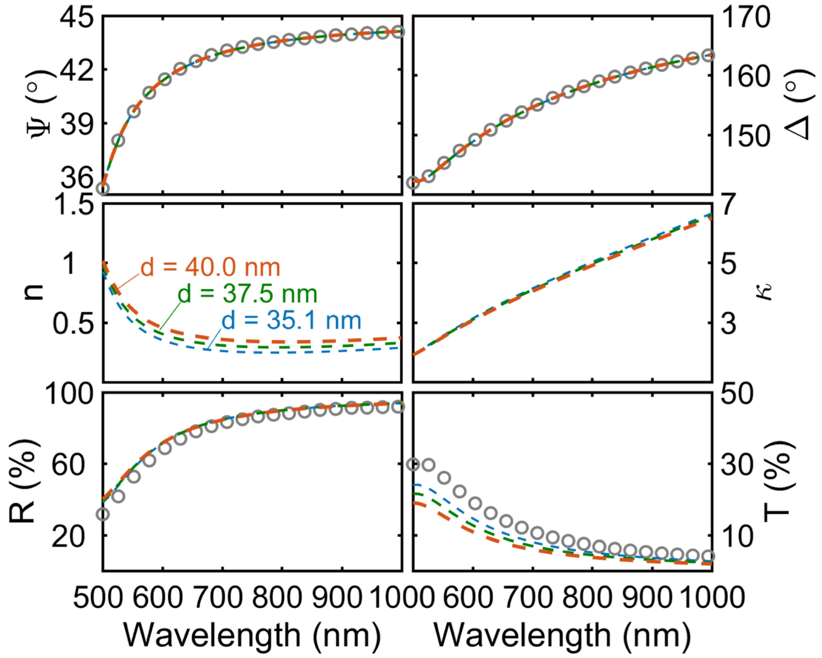

FIG. S1. **Fitting ambiguity in traditional techniques.** By fitting only (Ψ, Δ), EP4Model finds multiple sets of (n, κ, d), all of which reproduce (Ψ, Δ) perfectly following the forward functions $\mathbb{F}$ and $\mathbb{G}$ (the dashed lines in the first row), but none of them generated satisfactory (R, T) as shown in the third row. One must examine the goodness of fit on additional data, i.e., (R, T), in order to choose the best solution, which is $d = 35.1$ nm here. Such fitting ambiguity has also been reported in literature, such as Ref. [1] and references therein.

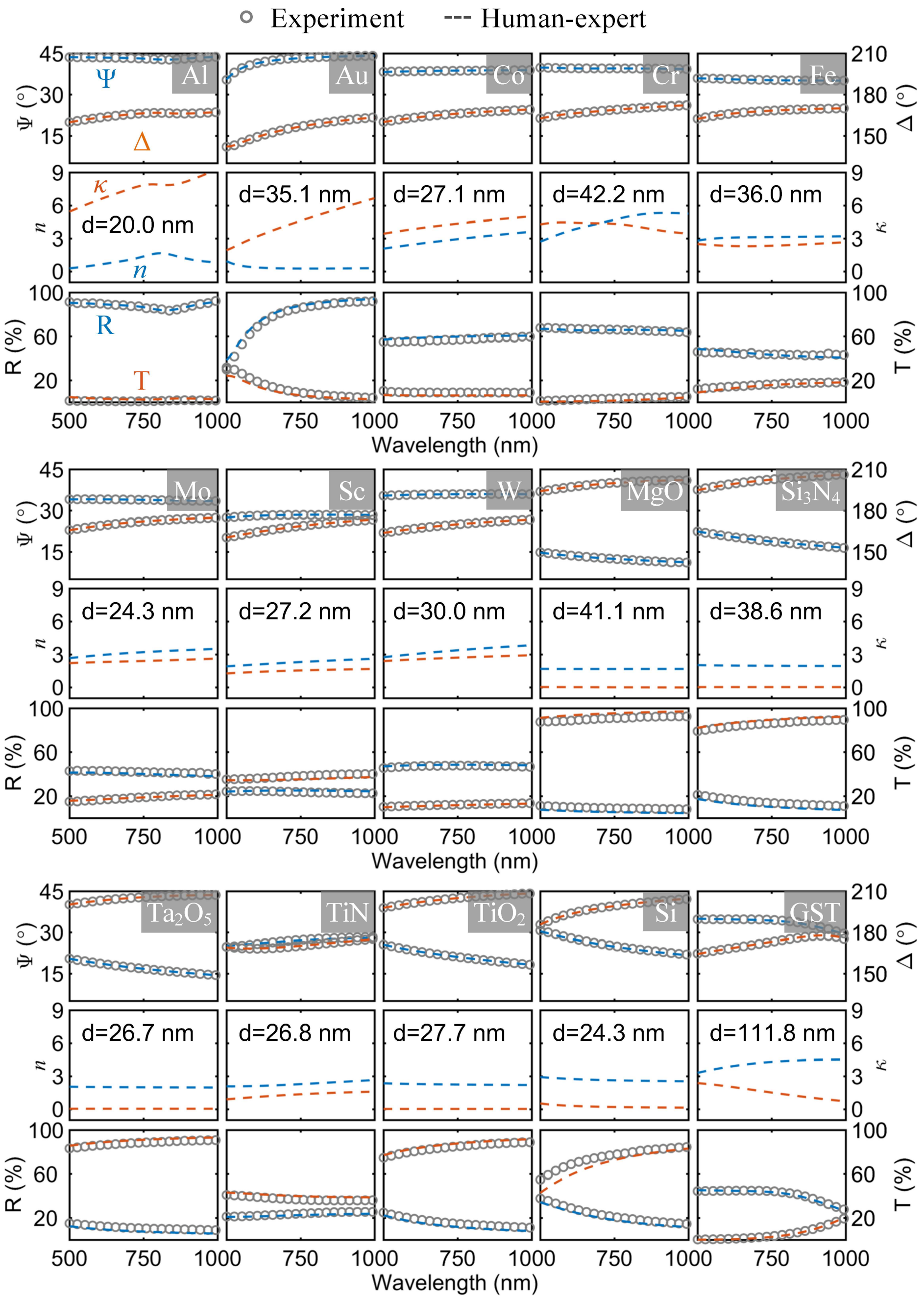

FIG. S2. **Results by traditional fitting techniques on various materials.** Experimentally measured (Ψ, Δ) and (R, T) are denoted as empty circles in the first and third rows of each panel, respectively. The model-generated ones are denoted as dashed lines correspondingly. Note that the traditional fitting technique only uses the experimental (Ψ, Δ) to derive (n, κ, d). And (R, T) data act as complements to choose the most suitable (n, κ, d) solution branch.

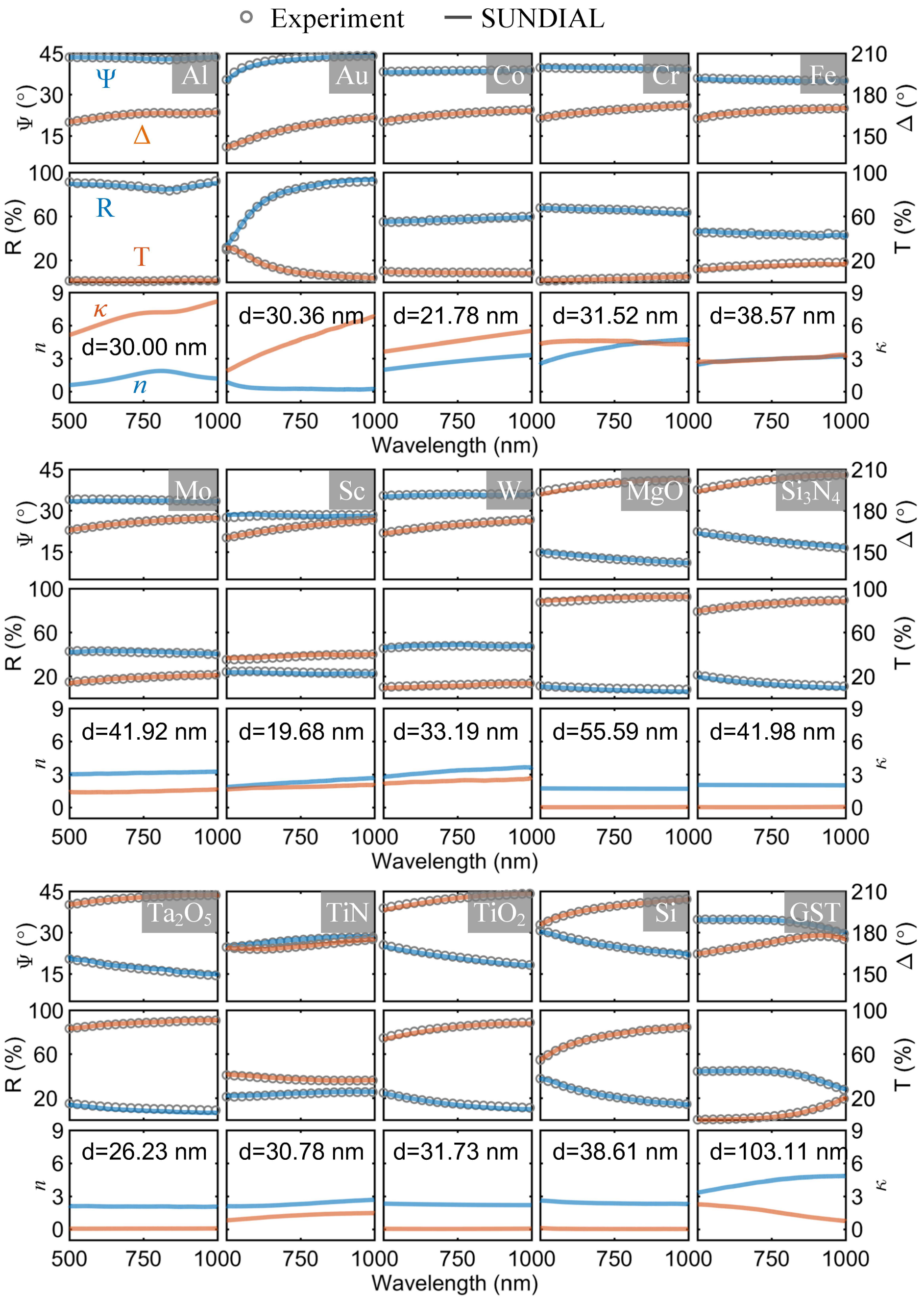

FIG. S3. **Results by SUNDIAL on various materials.** Experimentally measured (Ψ, Δ) and (R, T) are denoted by empty circles in the first and second rows of each panel, respectively. The model-generated ones are denoted as solid lines correspondingly. The SUNDIAL analyzes the experimental (Ψ, Δ, R, T) simultaneously to derive (n, κ, d).

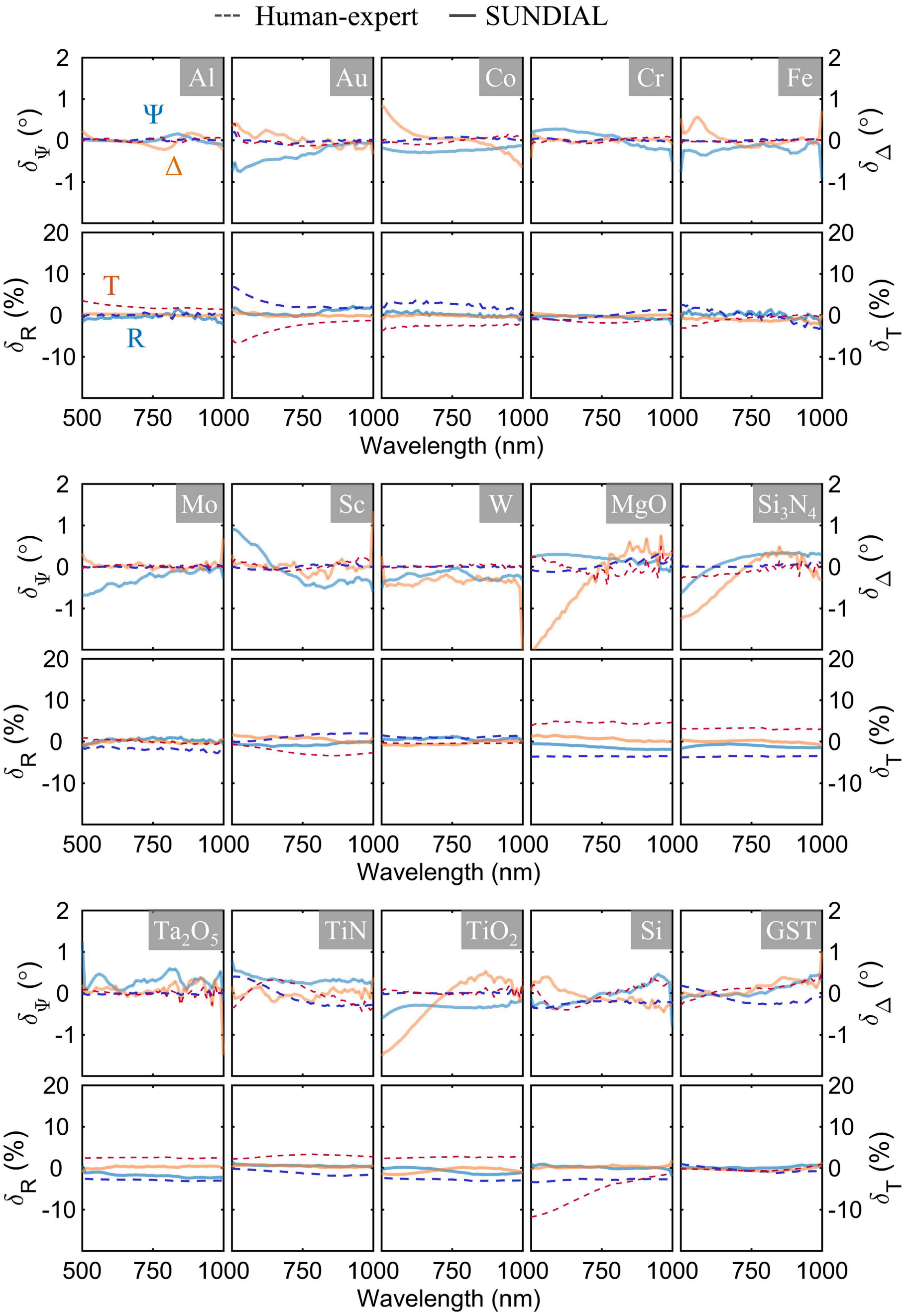

FIG. S4. **Comparison of SUNDIAL with traditional fitting technique.** Residuals δ of both methods (solid lines: the SUNDIAL, dashed lines: the traditional technique) on various materials are calculated and compared.

II. DETAILS OF THE SUNDIAL METHOD

A. Architecture of the inverse and forward networks

Table I shows details of features which compose input and target for forward and inverse networks of SUNDIAL. To be specific, $(n_{\text{film}}, \kappa_{\text{film}}, d_{\text{film}}, \theta, n_{\text{sub}}, \kappa_{\text{sub}}, n_{\text{air}}, \kappa_{\text{air}})$ and (Ψ, Δ, R, T) are input and output features for the forward networks which also act as the output and input features for the inverse networks.

TABLE I. Features for forward and inverse networks of SUNDIAL

**Feature**	**Description**
Ψ	Ellipsometric data
Δ	Ellipsometric data
R	Reflectance
T	Transmittance
n_{film}	Refractive index of interest
κ_{film}	Extinction coefficient of interest
d_{film}	Thickness of film
θ	Angle of incidence
n_{sub}	Refractive index of substrate (fused quartz here)
κ_{sub}	Extinction coefficient of substrate (fused quartz here)
n_{air}	Refractive index of air (= 1 here)
κ_{air}	Extinction coefficient of air (= 0 here)

There are four neural networks involved in the SUNDIAL, two (inverse and forward) for (Ψ, Δ) and two for (R, T). Each of them consists of three identical blocks of U-modules and additional input and output layers to accommodate to the input and target tensors, as shown in Fig. 2 in the main text and Fig. S5 here. Note that U-module is similar to the U-Net[2] except that a residual-style addition, instead of concatenation, has been used to combine features from both arms.

Neural network architecture is of great importance in many successful applications of deep convolutional neural networks. Milestones include the classical LeNet,[3] AlexNet,[4] ResNet,[5] U-Net,[2] etc. Novel architectures allow us train very deep neural networks and achieve remarkable performance. For the inverse and forward networks in the SUNDIAL framework, we propose to use this "stacked residual U-modules" architecture as backbones which may be viewed as a variant of U-Net. Our experiments showed that it outperformed others including ResNet by an order of magnitude which is consistent with the facts that for *dense regression* applications U-Net and its variants are usually the most effective architectures. Since in our case the tasks that both the inverse and forward networks tackle with are clearly dense (spectrum) regression, it is not totally unexpected that this stacked residual U-modules significantly outperformed other architectures.

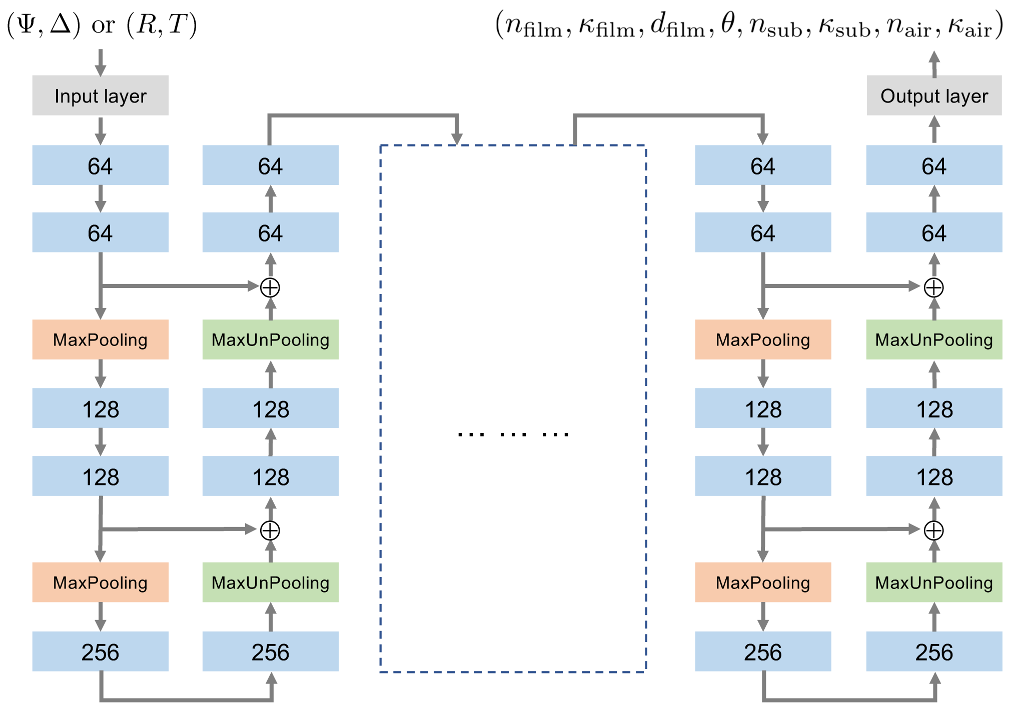

FIG. S5. **Architecture of inverse networks.** To have forward networks, we simply switch input features and targets as well as corresponding input and output layers. Kernel size of all convolutional layers is 3, except for the the input and output layers where the kernel size was set to 1.

B. Offline training on simulated data

To acquire domain/ellipsometric knowledge, the SUNDIAL is first trained offline on a large amount of simulated data which however may significantly deviate from the real-world experimental data. We applied data augmentation to increase the robustness of the trained model. The protocol is as follows: 1) Shift the input left or right randomly by a few wavelength which is not greater than 3 in our experiments; 2) Multiply the input features by a small random scale $(1+g*N_r)$, where g is a random number generated from a standard normal distribution, and N_r denotes the noise level not greater than 10^{-3} here.

To clearly illustrate the effect of data augmentation especially when the training data is not sufficient or does not contain the noise that is to be dealt with, we have conducted a series of experiments where a relatively small training set of 300 samples was used and enhanced with different amount of augmented samples, and plotted the accuracy (RMSE v.s. N_r) in Fig. S6. It can be seen that when data augmentation was applied, the error reduced by a significant amount. Heavier augmentation led to more significant reduction in error, until it reached a saturation point where the improvement between augmentation of $\times 20$ and augmentation of $\times 40$ was marginal. This demonstrated that the proposed data augmentation protocol is an effective tool to supplement the training dataset.

For both the inverse and forward networks, the training on simulated data has been done using ADAM[6] optimizer with learning rate 10^{-4} and weight decay 10^{-5}. We have also applied the cosine annealing strategy to change the learning rate. The batch size was 64 and the maximum epochs was set to 2000 with early stopping. The loss function were mean-square-error which has been widely used for regression.

C. Online inference on experimental data

To bridge the gap between training on the simulated data and inferring on the real-world data, we propose a novel iterative inference strategy based on stochastic gradient descent. Unlike conventional inference of computing outputs by a single forward pass on neural networks which is doomed to fail in our case, our proposed framework allows neural modules continue to adapt online on the real-world data until a satisfactory solution is obtained. The pseudo-code is shown in Algorithm 1.

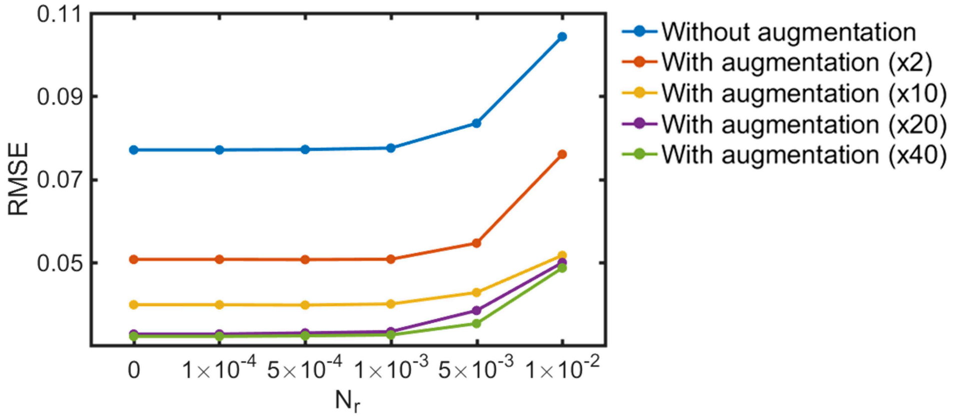

FIG. S6. **Accuracy v.s. N_r under different augmentation levels.** For M groups of training data, additional 2M, 10M, 20M and 40M augmented samples were generated respectively, denoted as with augmentation ($\times 2$, $\times 10$, $\times 20$, $\times 40$).

To create an augmented dataset $\mathcal{D}^{(t)}$ at the tth iteration, we first generate a number of random points in the neighbourhood of the current solution $x^{(t)}$ and then compute the corresponding pseudo-measurement using the forward functions. The protocol for random point generation is the same as data augmentation strategies outlined in the previous section for offline training, with an additional strategy: generating points that are close to the current solution along random directions. In our experiments, 100 points were generated following each data augmentation strategy and thus 300 points in total plus the current (center) solution were sampled to approximate the neighborhood of the current solution. By further training the forward networks on the augmented dataset, the forward networks can better approximate the forward functions in the neighborhood of the current solution.

Runtime analysis. The time complexity of the online inference is $\mathcal{O}\big(|\Theta_F| * K * N + |\Theta_I| * N\big)$ where K and N are defined in Algorithm 1. $|\Theta_F|$ and $|\Theta_I|$ are the size of the trainable weights of forward and inverse networks. In our implementation of SUNDIAL, the forward and inverse networks shared the same main architecture, therefore we have $|\Theta_F| = |\Theta_I| = S$, and the time complexity is $\mathcal{O}\big(S * K * N\big)$. In our experiments, K was set to 10, N was ranging from 50 to a few hundreds depending on materials to analyze, which corresponded to 15 minutes to 1.5 hours on a laptop with i7 CPU and NVIDIA GTX 1070 GPU (Mobile), and with the implementation of SUNDIAL which has not yet been optimized for speed.

It is worth noting that our method is designed to be built upon modern deep learning infrastructure and therefore can easily benefit, such as improvement of speed, from the

Algorithm 1: The SUNDIAL method

Input: An measurement (Ψ, Δ, R, T). The pretrained forward and inverse networks $\widetilde{F}_{\Theta_{F,\Psi\Delta}}$, $\widetilde{F}_{\Theta_{F,RT}}$, and $\widetilde{F}^{-1}_{\Theta_{I,\Psi\Delta}}$, $\widetilde{F}^{-1}_{\Theta_{I,RT}}$ with trainable weights $\Theta_{F,\Psi\Delta}$, $\Theta_{F,RT}$, $\Theta_{I,\Psi\Delta}$, and $\Theta_{I,RT}$. $\mathbb{F}, \mathbb{G}$ are the forward functions. α, β are step sizes. ϵ_1, ϵ_2 are predefined thresholds. γ is defined in Eq.(2) in the main text.

for $t = 0$ to N **do**

(a) Prepare an augmented dataset $\mathcal{D}^{(t)}$ for further training the forward network around the current solution

$$x^{(t)} = (n^{(t)}, \kappa^{(t)}, d^{(t)}) = \left(\widetilde{F}^{-1}_{\Theta^{(t)}_{I,\Psi\Delta}}(\Psi, \Delta) + \widetilde{F}^{-1}_{\Theta^{(t)}_{I,RT}}(R, T)\right)/2 \tag{1}$$

specifically, generate a number of random points in the neighborhood of $x^{(t)}$ and compute the corresponding pseudo-measurements

$$(\Psi_{D^{(t)}}, \Delta_{D^{(t)}}, R_{D^{(t)}}, T_{D^{(t)}}) = (\mathbb{F}(x^{(t)}), \mathbb{G}(x^{(t)})) \tag{2}$$

(b) Train the forward network on $\mathcal{D}^{(t)}$ for a number of iterations.

for $k = 0$ to K **do**

Update

$$(\Theta_{F,\Psi\Delta}, \Theta_{F,RT})^{(k+1)} = (\Theta_{F,\Psi\Delta}, \Theta_{F,RT})^{(k)} - \alpha \sum_{x \in \mathcal{D}^{(t)}} \frac{\partial\left(\gamma \left\|(\Psi_x, \Delta_x) - \widetilde{F}_{\Theta^{(t)}_{F,\Psi\Delta}}(x)\right\|_2 + (1-\gamma)\left\|(R_x, T_x) - \widetilde{F}_{\Theta^{(t)}_{F,RT}}(x)\right\|_2\right)}{\partial(\Theta_{F,\Psi\Delta}, \Theta_{F,RT})} \tag{3}$$

Stop if the residual r_1

$$\gamma \left\|(\Psi_x, \Delta_x) - \widetilde{F}_{\Theta^{(t)}_{F,\Psi\Delta}}(x)\right\|_2 + (1-\gamma)\left\|(R_x, T_x) - \widetilde{F}_{\Theta^{(t)}_{F,RT}}(x)\right\|_2 < \epsilon_1 \tag{4}$$

end

(c) Fix the weights Θ_F of the forward network, and run one step update of the whole framework using (Ψ, Δ, R, T) as both input and target. Only the weights of the inverse networks is updated in this step.

$$(\Theta_{I,\Psi\Delta}, \Theta_{I,RT})^{(t+1)} = (\Theta_{I,\Psi\Delta}, \Theta_{I,RT})^{(t)} - \beta \frac{\partial\left(\gamma \left\|(\Psi, \Delta) - \widetilde{F}_{\Theta^{(t)}_{F,\Psi\Delta}}(x^{(t)})\right\|_2 + (1-\gamma)\left\|(R, T) - \widetilde{F}_{\Theta^{(t)}_{F,RT}}(x^{(t)})\right\|_2\right)}{\partial(\Theta_{I,\Psi\Delta}, \Theta_{I,RT})} \tag{5}$$

(d) Stop if the residual r_2

$$\gamma \left\|(\Psi, \Delta) - \mathbb{F}(x^{(t)})\right\|_2 + (1-\gamma)\left\|(R, T) - \mathbb{G}(x^{(t)})\right\|_2 < \epsilon_2$$

end

Output: $n^*, \kappa^*, d^* = \left(\widetilde{F}^{-1}_{\Theta^*_{I,\Psi\Delta}}, \widetilde{F}^{-1}_{\Theta^*_{I,RT}}\right)(\Psi, \Delta, R, T)$ where $\left(\widetilde{F}^{-1}_{\Theta^*_{I,\Psi\Delta}}, \widetilde{F}^{-1}_{\Theta^*_{I,RT}}\right)$ is the inverse networks with the smallest residual r_2.

advancing of the computing hardware and software with very little modification of the method itself.

Our code is available on https://github.com/Chaoscendence/sundial.

D. Robustness of the trained models

We investigate the robustness, i.e., how the noise in (Ψ, Δ, R, T) affects the accuracy of the SUNDIAL. Since in the ellipsometric problem one cannot know the (n, κ, d) beforehand, we adopt the approach of using synthetic/simulated data. We took 500 groups of (n, κ, d) and corresponding synthetic (Ψ, Δ, R, T) from the test (sub-)set of the simulated dataset for offline training where all the (n, κ, d) come from the Palik and Sopra databases. With this setting, we were able to calculate the model accuracy. It is worth noting that these 500 samples were not used for training, but testing the performance of the SUNDIAL on the simulated data, as we have followed the standard protocol of randomly partitioning the whole simulated dataset (6240 samples) into non-overlapping training (5240 samples), validation (500 samples) and test sets (500 samples). To simulate data with different levels of noise, (Ψ, Δ, R, T) was multiplied by $(1 + g * N_r)$, where g is a random number generated from a standard normal distribution, and N_r denotes the noise level. We plot the RMSE of (n, κ, d) with respect to different levels of N_r in Fig. S7. It can be seen that the SUNDIAL shows good accuracy on simulated data when $N_r < 10^{-3}$, to be specific, about 5×10^{-3}, 3×10^{-3}, and 0.8 nm for RMSE of (n, κ, d), respectively. On the other hand, as the (Ψ, Δ, R, T) becomes too noisy with N_r of 10^{-2}, the RMSE of (n, κ, d) increases.

E. Noise in experimental data

The experimentally measured (Ψ, Δ) and (R, T) data would inevitably contain noise (from for example power fluctuation of light source, dark noise of detector of ellipsometer and spectrometer, etc). In Fig. S8(a), we show the noise σ of the measured (Ψ, Δ, R, T) for different materials. The noise of the measured (Ψ, Δ, R, T) is calculated by $\sigma = \sqrt{\frac{1}{N}\sum_{i=1}^{N}\left(\frac{S_i^{Exp} - \hat{S}_i^{Exp}}{\hat{S}_i^{Exp}}\right)^2}$, in which S is Ψ, Δ, R, or T. N is the number of wavelength points included in each spectrum. S_i^{Exp} and $\hat{S}_i^{Exp}$ present the measured raw data and the ones after smoothing to filter out the noise, respectively (a typical example of

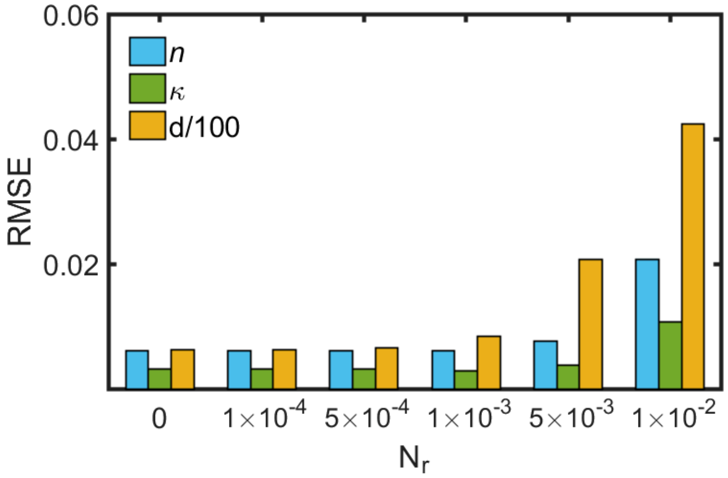

FIG. S7. **Accuracy of SUNDIAL on noise levels (N_r) of (Ψ, Δ, R, T) datasets.**

S_i^{Exp} and $\hat{S}_i^{Exp}$ for Si is shown in Fig. S8(b)). In practice, we may choose γ according to the relative importance, e.g. noise level, of (Ψ, Δ) and (R, T). However, both of (Ψ, Δ) and (R, T) show quite small noise in our case (indicated by dashed lines, below 6×10^{-4}), we do not have strong evidence to favor any of them. Thus we suggest $\gamma = 0.5$ in our study to equilibrate the contributions from both the (Ψ, Δ) and (R, T) and indeed it showed good results in our experiments (shown by Fig. 4(c) in main text).

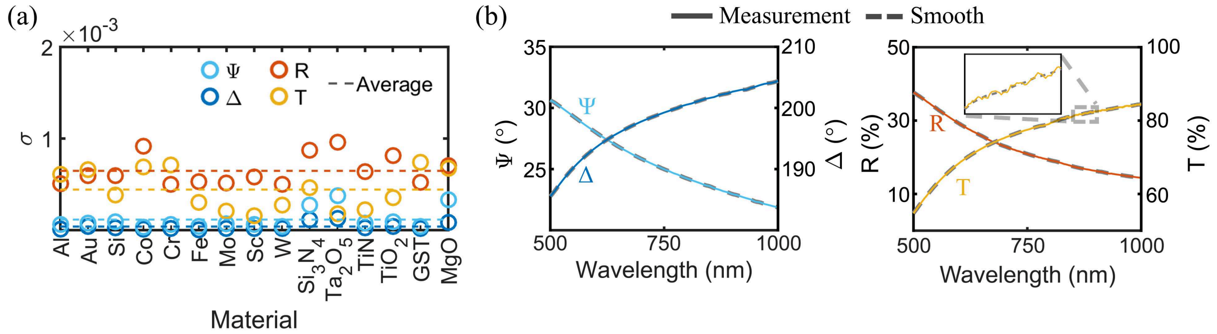

FIG. S8. **Noise in experimental data.** **(a)** σ levels of measured (Ψ, Δ, R, T) for different materials. Dashed lines present average noise levels. **(b)** Experimentally measured and smoothed (Ψ, Δ, R, T) spectra. Results of Si are given here as an example. Solid lines are raw data, and dashed lines are ones after smoothing. Detailed comparison between the raw and the smooth data is inset in right panel.

REFERENCES

[1]H. G. Tompkins and J. N. Hilfiker, *Spectroscopic Ellipsometry: Practical Application to Thin Film Characterization* (Momentum Press, 2015).

[2]O. Ronneberger, P. Fischer, and T. Brox, "U-net: Convolutional networks for biomedical image segmentation," in "Med. Image. Comput. Comput. Assist. Interv.", (Springer, 2015), pp. 234–241.

[3]Y. LeCun, L. Bottou, Y. Bengio, and P. Haffner, "Gradient-based learning applied to document recognition," Proc. IEEE **86**, 2278–2324 (1998).

[4]A. Krizhevsky, I. Sutskever, and G. E. Hinton, "Imagenet classification with deep convolutional neural networks," in "Adv. Neural. Inf. Process. Syst.", (2012), pp. 1097–1105.

[5]K. He, X. Zhang, S. Ren, and J. Sun, "Deep residual learning for image recognition," in "Proc. IEEE. Comput. Soc. Conf. Comput. Vis. Pattern Recognit.", (2016), pp. 770–778.

[6]D. P. Kingma and J. Ba, "Adam: A method for stochastic optimization," arXiv preprint arXiv:1412.6980 (2014).
